# Decreased Effective Macromolecular Crowding in Escherichia coli Adapted to Hyperosmotic Stress

**DOI:** 10.1128/JB.00708-18

**Published:** 2019-04-24

**Authors:** Boqun Liu, Zarief Hasrat, Bert Poolman, Arnold J. Boersma

**Affiliations:** a Department of Biochemistry, Groningen Biomolecular Sciences and Biotechnology Institute, University of Groningen, Groningen, The Netherlands; b Jilin Provincial Key Laboratory of Nutrition and Functional Food, Jilin University, Changchun, People's Republic of China; c Zernike Institute for Advanced Materials, University of Groningen, Groningen, The Netherlands; d DWI-Leibniz Institute for Interactive Materials, Aachen, Germany; Queen Mary University of London

**Keywords:** FRET-based sensors, biochemical organization of cytoplasm, energy status, excluded volume, macromolecular crowding, osmotic stress

## Abstract

Escherichia coli adapts to changing environmental osmolality to survive and maintain growth. It has been shown that the diffusion of green fluorescent protein (GFP) in cells adapted to osmotic upshifts is higher than expected from the increase in biopolymer volume fraction. To better understand the physicochemical state of the cytoplasm in adapted cells, we now follow the macromolecular crowding during adaptation with fluorescence resonance energy transfer (FRET)-based sensors. We apply an osmotic upshift and find that after an initial increase, the apparent crowding decreases over the course of hours to arrive at a value lower than that before the osmotic upshift. Crowding relates to cell volume until cell division ensues, after which a transition in the biochemical organization occurs. Analysis of single cells by microfluidics shows that changes in cell volume, elongation, and division are most likely not the cause for the transition in organization. We further show that the decrease in apparent crowding upon adaptation is similar to the apparent crowding in energy-depleted cells. Based on our findings in combination with literature data, we suggest that adapted cells have indeed an altered biochemical organization of the cytoplasm, possibly due to different effective particle size distributions and concomitant nanoscale heterogeneity. This could potentially be a general response to accommodate higher biopolymer fractions yet retaining crowding homeostasis, and it could apply to other species or conditions as well.

**IMPORTANCE** Bacteria adapt to ever-changing environmental conditions such as osmotic stress and energy limitation. It is not well understood how biomolecules reorganize themselves inside Escherichia coli under these conditions. An altered biochemical organization would affect macromolecular crowding, which could influence reaction rates and diffusion of macromolecules. In cells adapted to osmotic upshift, protein diffusion is indeed faster than expected on the basis of the biopolymer volume fraction. We now probe the effects of macromolecular crowding in cells adapted to osmotic stress or depleted in metabolic energy with a genetically encoded fluorescence-based probe. We find that the effective macromolecular crowding in adapted and energy-depleted cells is lower than in unstressed cells, indicating major alterations in the biochemical organization of the cytoplasm.

## Abstract (precis)

Bacteria adapt to ever-changing environmental conditions such as osmotic stress and energy limitation. It is not well understood how biomolecules reorganize themselves inside Escherichia coli under these conditions. An altered biochemical organization would affect macromolecular crowding, which could influence reaction rates and diffusion of macromolecules. In cells adapted to osmotic upshift, protein diffusion is indeed faster than expected on the basis of the biopolymer volume fraction. We now probe the effects of macromolecular crowding in cells adapted to osmotic stress or depleted in metabolic energy with a genetically encoded fluorescence-based probe. We find that the effective macromolecular crowding in adapted and energy-depleted cells is lower than in unstressed cells, indicating major alterations in the biochemical organization of the cytoplasm.

## INTRODUCTION

The environment induces changes in the internal organization of a cell, for example, during nutrient depletion or osmotic changes. Nutrient depletion halts the diffusion of 100-nm particles (1), while osmotic stress decreases the diffusion of 10-nm proteins (2, 3). Cells adapt to environmental stresses to resume growth, but their internal structure may be changed. For instance, the lateral diffusion coefficient of green fluorescent protein (GFP) in cells adapted to osmotic stress is higher than expected from the biopolymer volume fraction (4). The cytoplasmic structure, or biochemical organization, can influence GFP diffusion in various ways by changing, for example, the microscopic viscosity, the association of proteins with (non)specific binding partners, the presence of barriers such as membrane invaginations, or sieving effects by, for example, the nucleoid or transertions (2, 4–6). Thus, the change in diffusion indicates that the biochemical organization has undergone a change, but the nature of this transition is not well understood.

One important aspect of cell physiology is macromolecular crowding (7–10), which influences lateral diffusion but also induces excluded volume effects. The steric repulsion between biomacromolecules (“crowders”) at a high concentration reduces the configurational entropy of these crowders. A biochemical reaction (“tracer”) that takes place in this environment reduces its volume to maximize the configurational entropy for the crowders. This effect can especially drive large complexes together where crowders are excluded from the volumes between the tracers (overlap volumes). In this manner, the excluded volume provides an additional force to sort biomolecules to provide biochemical organization by favoring (supra)molecular complexes and membraneless compartments. Excluded volume effects are increased, for example, (i) when the crowders are actually mobile and can increase their translational degrees of freedom, and (ii) when they are smaller than the tracer (11). Thus, immobile crowders and/or crowders that are larger than the tracer display less of the crowding effect in the classical sense but can induce confinement, where the shape of the confinement enforces a shape onto the tracer, instead of the minimum volume obtained in classical crowding. Macromolecular crowding, or excluded volume effects, therefore does not necessarily correlate with the biopolymer volume fraction.

Macromolecular crowding is one of the main parameters that changes when the volume of a cell changes, which can occur when the medium osmolality changes. Upon osmotic upshift, the cells immediately shrink, and many cells counteract this by taking up potassium ions, which is best documented for Escherichia coli (12–14). Subsequently, E. coli synthesizes or takes up available compatible solutes and adjusts the proteome to adapt to the osmotic upshift. Researchers showed that in E. coli, over 300 genes are up- or downregulated by osmotic upshift (15–17). E. coli further increases its RNA/protein ratio due to an increase in ribosome content when adapted to high osmotic strength (18), possibly to compensate for the decreased rate of translation.

We apply here a set of fluorescence resonance energy transfer (FRET)-based sensors that enable the quantification of macromolecular crowding during adaptation to osmotic stress. The sensors have shown excellent performance in quantifying crowding during osmotic stress in mammalian cells (19–21) and allow a detailed analysis of crowding in the bacterium Escherichia coli (19, 22). The sensors vary in size, with crGE being the largest probe, with a linker region that contains two α-helices and three random coils between the fluorescent proteins that form a FRET pair (mCerulean3 as the donor and mCitrine as the acceptor). The crE6G2 sensor contains a linker with two α-helices and a small random coil, while the crG18 probe contains a single long random coil.

Using these probes, we show here that macromolecular crowding increases upon osmotic upshift and returns within 2 to 5 h to a level lower than the crowding prior the osmotic shift. We explain the lower effective excluded volume by the hypothesis that the biochemical organization of the cytoplasm is significantly altered, with components that exert fewer excluded volume effects for molecules in the size range of our molecular probes.

## RESULTS

### Macromolecular crowding decreases after an osmotic upshift.

**FIG 1:**
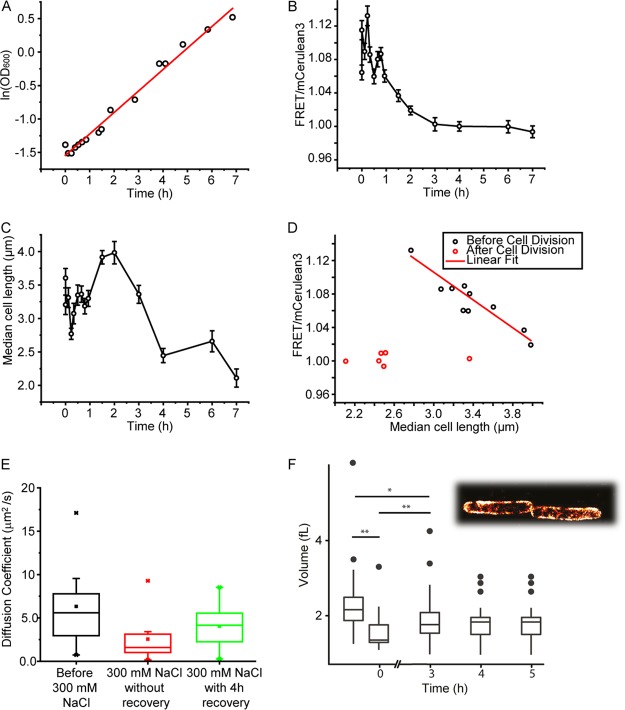
Response of *E. coli* BL21(DE3) containing the crGE probe in pRSET A to the addition of 300 mM NaCl. (A) The ln(OD_600_) decreases after the upshift and subsequently increases linearly over time (passing the preupshift OD_600_ after ∼1 h). The OD_600_ is corrected for continuous dilution of the culture to maintain the OD_600_ between 0.1 and 0.3. The data fit a linear curve with an *R*^2^ value of 0.99, indicating exponential growth throughout the course of the experiment. (B) The FRET/mCerulean3 ratio of the crGE probe as measured by confocal fluorescence microscopy. The ratios immediately increase after osmotic upshift and decrease after 1 h to levels lower than prior to the osmotic upshift. All data are for at least 60 *E. coli* cells, with a FRET/mCerulean3 standard deviation of ∼0.05 and a standard error of ∼0.009. (C) Osmotic upshift results in a decrease in median cell length as measured by fluorescence microscopy (same cells as in panel B), which is followed by an increase in length of the synchronized cells until division starts, resulting in smaller cells than those under preupshift conditions. (D) Data from panels B and C combined showing the relation (linear approximation, *R*^2^ = 0.82) between the FRET/mCerulean3 ratio and the median cell length (black circles), which holds until the cells divide. After that, the FRET/mCerulean3 ratio remains low (red circles), which is after 3 h in panels B and C. (E) Lateral diffusion of the crGE probe in unstressed and 300 mM NaCl stressed and adapted cells. The FRAP measurements were carried out as described previously (38). Displayed are the box plots generated for measurements of 10 to 20 cells, each from the same culture to allow comparison. The box represents 25 to 75% of the data range, whiskers are within the 1.5 interquartile range, the bar in the box is median, the square is the average, and stars are outliers. (F) Cell volume changes during hyperosmotic stress. *E. coli* BL21(DE3) expressing LacY-YPet was used, and the contours from single-molecule localizations by PALM were used to obtain the volumes of the cells (see inset). Untreated cells are measured at *t* of −1 h in MOPS-glucose; to capture the data point at *t* of 0 h, the cells were resuspended in MOPS medium without potassium and glucose to prevent recovery and subsequently treated with 300 mM NaCl. For time points 3, 4, and 5 h, cells were left to adapt to 300 mM NaCl in regular MOPS-glucose medium. For each data point, ∼30 cells were imaged and analyzed (*, *P* < 0.05; **, *P* < 0.005; paired sample *t* test).

To determine the crowding during adaptation to an osmotic upshift, we added 300 mM NaCl to exponentially growing E. coli BL21(DE3) cells and allowed the cells to adapt to the increased medium osmolarity. To monitor the macromolecular crowding, we expressed the crGE probe under leaky expression of the T7 promoter, which prevents maturation artifacts, as we described previously (23). To compare our results with literature data, we performed the experiments in morpholinepropanesulfonic (MOPS)-glucose medium (2, 4, 18). We find that under these conditions, the osmotic upshift initially decreases the optical density at 600 nm (OD_600_) of the cell culture, which slowly recovers to preupshift levels over about an hour. After this, the cultures maintain a steady growth rate throughout the experiments (Fig. 1A).

We took samples from the main culture for analysis by confocal fluorescence microscopy, excited the crGE probe at 405 nm, and determined the emission between 450 and 505 nm for mCerulean3 and between 505 and 795 nm for the FRET channel, as described previously (19). Before the upshift, the FRET/mCerulean3 ratio is a mean ± standard error of the mean (SEM) of 1.06 ± 0.009 (*n* = 98; SD = 0.07), which immediately increases to a mean ± SEM of 1.12 ± 0.01 (*n* = 98) upon the addition of 300 mM NaCl (Fig. 1B). These FRET/mCerulean3 ratios are equivalent to 21% (wt/wt) and 30% (wt/wt) Ficoll, respectively (19). The FRET/mCerulean3 ratio follows the OD_600_ by returning to the preupshift level within 1 h. After this, the FRET/mCerulean3 ratio decreases further to a mean ± SEM of 1.00 ± 0.006 (*n* = 90) over an additional 1 to 2 h, where it remains for at least 23 h. This FRET/mCerulean3 ratio is equivalent to 13% (wt/wt) Ficoll (19). We maintain the cells in the exponential phase of growth by continuously refreshing the medium. The observations are similar for the crG18 sensor that contains a different linker (see Fig. S1 in the supplemental material) (22). We find that the addition of 100 mM NaCl does not lead to significant changes, while the addition of 500 mM NaCl provides a decrease similar to that with 300 mM NaCl (Fig. S2). This apparent threshold coincides with the occurrence of membrane invaginations when 300 and 500 mM NaCl are added, which do not occur with 100 mM NaCl (see also references 2 and 24).

To confirm that the ratiometric FRET reports genuine changes in excluded volume, rather than, e.g., binding of specific molecules to the sensors, we performed a series of control experiments. We investigated the influence of cell lysate on purified sensor (Fig. S3). We lysed E. coli strains with or without 300 mM NaCl and did not find an effect of the cell-free lysate of 7 mg of total protein/ml on purified crG18. Hence, cytoplasmic macromolecules do not specifically interact with the sensors. The same applies for small molecules (metabolites and osmolytes) that are abundant in E. coli (19, 22, 25). To confirm that the probes are not truncated in osmotically stressed cells, we performed SDS-PAGE analysis. The gels show intact probes in control and osmotically stressed cells (Fig. S4). To assess whether the mCitrine fluorescence is not quenched by acidification of the cells during adaptation, we excited the mCitrine directly at 488 nm, and we did not find a decrease in intensity (Fig. S5). To show that the ratiometric FRET signal is independent of the maturation of the fluorescent proteins (23), we exchanged the mCerulean3 with the faster-maturing mTurquoise2 (crTC2) and obtained a qualitatively similar readout (Fig. S6). Also, changing the acceptor to cpmVenus (crcpGE) did not lead to a different result, excluding effects specific to the fluorescent proteins.

Next, we benchmarked the diffusion of the probes against GFP under conditions of osmotic stress and adaptation by fluorescence recovery after photobleaching (FRAP) (Fig. 1E). The median diffusion coefficient decreases from 5.6 (±1.6 [standard deviation]) μm^2^/s to 1.6 (±1.3) μm^2^/s and subsequently increases 4 h after the addition of NaCl to 4.2 (±0.3) μm^2^/s. The changes in diffusion do not reflect the FRET of the sensor precisely, likely because factors such as immobile barriers influence diffusion differently (26). These diffusion coefficients compare to 14.1 (±3.8), 1.7 (±1.1), and 10.3 (±3.1) μm^2^/s, respectively, for the diffusion of GFP (4). The last data value was obtained at 1.02 osM, while our osmolarity cumulates to 0.88 osM (the osmolarity of MOPS medium plus 300 mM NaCl). The difference in diffusion coefficients of crGE and GFP corresponds with the differences in sizes, but, importantly, the relative changes in mobility indicate that they probe similar biochemical organizations of the cytoplasm.

### Macromolecular crowding relates to cell length until cells divide.

Macromolecular crowding should relate to the volume of a cell when the number of inert biomacromolecules is constant. To investigate whether the decrease in crowding indeed relates to volume changes and cell growth, we determined the cell length and volume during adaptation. To estimate the volume of the cells, we performed photoactivated localization microscopy (PALM) measurements using the inner membrane protein LacY fused to YPet. We find that the volume decreases by 30% immediately after adding 300 mM NaCl (Fig. 1F), while 3 h after the osmotic upshift, the volume recovered to ∼1.8 fl, which is 82% of the value before the upshift. When measuring the length of the cells from brightfield images by confocal microscopy (Fig. 1C), we find that cells immediately become shorter by 20% upon addition of 300 mM NaCl, and the length returns to the value before the osmotic upshift after 30 min. After 60 to 90 min, the average cell length starts to increase. Afterwards, when most cells divide (doubling time of 2 h), apparently in a synchronized manner, the average cell length decreases. After this, the average length remains short in the adapted cells. The decrease in length is more pronounced than the decrease in volume, which implies that the adapted cells have an increased diameter. Yet, the overall trend is similar for both cell length and volume. We find that the crowding relates reciprocally with cell length and cell volume (Fig. 1D). After ∼2 h, which coincides with the moment the cells divide, the relationship between crowding and cell volume no longer holds. We find these trends for both the crGE and the crG18 (Fig. S1). Hence, the results indicate that the crowding, as anticipated, is proportional to the cell volume after the osmotic upshift, but the relationship changes when cells adapt to the osmotic stress.

### Elongation or division is not needed to change crowding.

To assess whether cell elongation or division is strictly correlated with crowding during adaptation to osmotic stress, we studied individual cells in microfluidics devices. This allows comparison of cells that adapt to those that do not, and we can dissect whether or not cell division influences the levels of crowding. E. coli cells growing in 0.1× MOPS-glucose medium (supplemented with 160 mM NaCl) in microfluidic devices can be analyzed for at least 6 h (Fig. S7). We observed less fluctuation in apparent crowding when cells grew in 0.1× MOPS-glucose than when they grew in undiluted MOPS-glucose medium, a finding for which we do not have an explanation. Exogenous MOPS accumulates in E. coli (27) and may disturb its physiology. We found that the growth rates in 0.1× MOPS-glucose and MOPS-glucose medium are similar in liquid culture, ∼0.5 h^−1^. Hence, for all the experiments in the microfluidic chamber, we incubated the cells in 0.1× MOPS-glucose medium supplemented with 160 mM NaCl to obtain the same osmolarity as MOPS-glucose medium.

We incubated the cells in the microfluidic chamber for 2 h, after which we replaced the medium with 0.1× MOPS-glucose with 460 mM NaCl (hence, 300 mM extra). To confirm that crowding changes are independent of the type of crowding sensor, we compared the FRET signals of crGE, crE6G2, and crG18 during the osmotic upshift. We find a similar decrease in ratiometric FRET as observed in the experiments in batch culture. We further applied an osmotic upshift with sorbitol, showing a response similar to that with equiosmolar amounts of sodium chloride (Fig. S8). We counted the cells and noted that the cell number increased steadily until the osmotic upshift, after which the osmotic stress reduced the increase temporally (Fig. S9).

**FIG 2:**
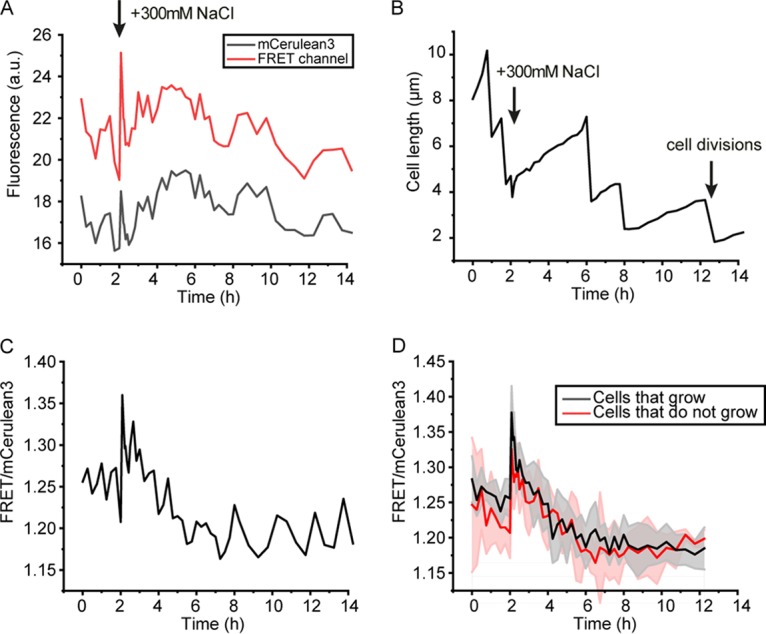
Single-cell analysis in microfluidics, monitored by confocal microscopy. At 2 h, the medium flown into the chamber that holds the cells was changed from 0.1× MOPS plus 160 mM NaCl to 0.1× MOPS plus 460 mM NaCl (net increase, 300 mM NaCl). The *E. coli* BL21(DE3) cells contained the crE6G2 sensor in pRSET A. (A) Fluorescence intensity of a single cell over time; the emissions from the mCerulean3 and the FRET channel are shown. (B) Cell length of the same cell analyzed in panel A, showing elongation and cell division and a small transient decrease in cell length following the osmotic upshift at 2 h. The time between cell divisions varies significantly. (C) The FRET/mCerulean3 ratio of the same cell, showing a qualitatively similar time course of the crowding as in the batch experiments. (D) Average of the population of cells that grow after osmotic upshift (*n* = 9) compared with cells that do not grow (*n* = 4). Shaded areas are the corresponding standard deviations.

We previously found that of the three probes, crE6G2 was most sensitive to changes in macromolecular crowding (22). Single-cell analyses provide a significant amount of noise (Fig. 2A and C), yet for most cells, the trend in the FRET/mCerulean3 ratio could clearly be distinguished with crE6G2, allowing a comparison of crowding with cell length and division time (Fig. 2B). The single cells showed a transient decrease in length in the first 5 min after osmotic upshift, which coincides with the presence of membrane invaginations. After 5 to 10 min, the cells started to elongate again. Upon the addition of 300 mM NaCl, the shape of the FRET/mCerulean3 response of individual cells is similar to that of ensemble measurements. Although the single-cell FRET/mCerulean3 data are noisy, we infer that cell division is not strictly correlated with the macromolecular crowding, in that cell division appears to be rather stochastic and unrelated to the FRET/mCerulean3 curve, which is similar for most of the cells. Furthermore, when we compiled data of cells that grew and compared them to data of those that did not grow, we did not observe a significant difference in crowding levels (Fig. 2D). In both cases, we found the decrease to be significant (*P* < 0.05, Student’s *t* test). Hence, elongation and division do not necessarily drive the decrease in crowding upon adaptation to 300 mM NaCl, but they coincide with the crowding changes on the population level.

### Energy decoupling also decreases crowding.

Next, we determined the crowding in cells that were deprived of energy by using a protonophore [carbonyl cyanide-4-(trifluoromethoxy)phenylhydrazone (FCCP)] to dissipate the electrochemical proton gradient and thereby deplete the cells of ATP. Energy-depleted E. coli cells undergo a transition in their internal organization that hampers the diffusion of 100-nm but not 10-nm particles (1). Our probes are in the 10-nm size range, that is, our probes behave as a disordered protein with a distance between the centers of the fluorescent proteins of ∼5 to 10 nm. Therefore, if crowding would be the only factor influencing diffusion, 10-nm particles should not experience a change in crowding because their diffusion does not change. If, on the other hand, native biomacromolecules are assembled into larger structures, e.g., resulting in a transition of the cytoplasm from a fluid into a more solid-like “colloidal glassy” state (1, 28), an inert 10-nm particle could experience less crowding. Such a state could be enhanced by the depletion of ATP, because ATP has been implicated as biological hydrotrope to enhance the solubility of proteins (29).

**FIG 3:**
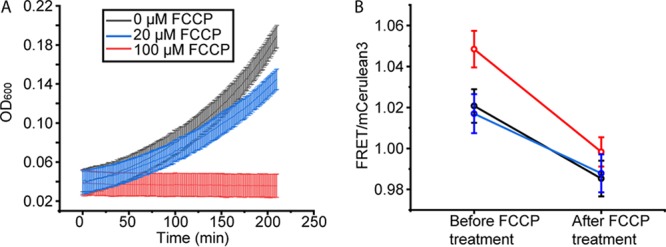
Crowding of energy-depleted *E. coli* as probed by crGE. (A) The effect of FCCP on the growth of *E. coli* BL21(DE3) in MOPS minimal medium supplemented with glucose. Error bars are from four technical repeats. (B) Application of 100 μM FCCP results in an immediate drop in the FRET/mCerulean3 ratio. Three independent biological repeats are displayed; error bars are error in the fit of FRET versus mCerulean3 intensity over about 100 cells.

Because the effectiveness of protonophores depends on various factors (e.g., membrane concentration, E. coli strain, carbon source, and medium pH), we first assessed the FCCP concentration required to halt cell growth and found that 100 μM was necessary under our experimental conditions (Fig. 3A). Next, we applied FCCP to exponentially growing E. coli cells that contained the crGE probe and measured the FRET/mCerulean3 ratios (Fig. 3B). The measurements were performed within 2 min after the addition of FCCP. We find that the FRET/mCerulean3 ratio drops upon the addition of FCCP and reaches values comparable to those of cells adapted to 300 mM NaCl. We thus conclude that the effective excluded volumes probed by crGE of 300 mM NaCl-adapted and energy-depleted cells are similar.

## DISCUSSION

We have used previously developed crowding sensors to probe changes in the excluded volume of E. coli cells upon osmotic stress and energy depletion. We show that the effective excluded volume of E. coli increases upon osmotic upshift but subsequently decreases to values below those of unstressed cells. We find that in the first 1 to 2 h, the changes in crowding relate in a reciprocal manner to the cytoplasmic volume. When cells adapt to osmotic upshift conditions (300 mM NaCl), the apparent crowding levels become lower than those of unstressed cells.

**FIG 4:**
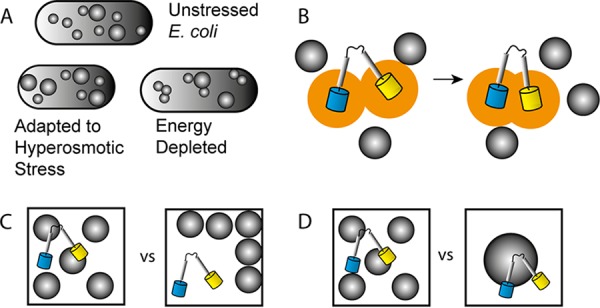
Changes in biochemical organization that affect crowding in cells. (A) Adaptation to osmotic stress and energy depletion changes both the size and spatial distribution of the macromolecules. (B) Working mechanism behind probe compression, where the excluded volume (orange) decreases due to crowding. This is caused by (i) increasing the translational degrees of freedom for the crowders and (ii) an osmotic pressure difference (depletion force) between the bulk and the crowder-inaccessible volume within the probe. (C) Immobile crowders do not affect the behavior described in panel B. Additionally, spatial heterogeneity increases the distance between probe and crowder and reduces the frequency of collision. (D) At a similar volume fraction, smaller crowders provide more entropy gain by virtue of number density and larger osmotic pressure differences.

Simply based on the biopolymer volume fraction, one would expect an increased macromolecular crowding in cells that have adapted to growth at increased osmolarities. Yet, Konopka et al. already showed for the first time that the diffusion of GFP was too high compared to what was expected from the biopolymer volume fraction (4). This in combination with our result confirms that the biopolymer volume fraction is not the sole determinant of crowding effects, which can be expected from theory (8) and suggests that there is a structural change in the cytoplasm (Fig. 4). Indeed, the Hwa group recently showed that cells adapted to hyperosmotic stress have a higher ribosome-to-overall-protein ratio than do cells before osmotic stress (18). This could explain the altered dependence on the volume fraction; ribosomes are about 20 nm in diameter, while our probes contain a disordered domain and are in the range of 5 to 10 nm and thus should exert less of a classical excluded volume effect than that of a smaller protein crowder. Moreover, if the ribosome is attached to mRNA, its effective size is much larger and it would not diffuse freely, which is needed for classical crowding effects. The Holt group recently investigated the size dependence of crowding induced by ribosomes in yeast cells (30) and showed that an increased ribosome concentration reduced the lateral diffusion of particles in a size-dependent manner, as 40-nm particles had a lower diffusion coefficient than did 20-nm particles, while the diffusion of 5-nm particles was not influenced. Thus, an increase in ribosome-to-overall-protein ratio at the same biopolymer volume fraction would diminish the crowding effect.

The reciprocal relationship between cell volume and crowding during the first 1 to 2 h after the osmotic upshift shows that macromolecular crowding behaves in a manner that one would expect from concentrating and diluting a solution of inert crowders. After this, or concomitantly, a major change in the biochemical transition may occur, and the relationship between cell volume and apparent crowding no longer holds. We cannot make a precise comparison of these time scales with literature data given that the media, strain, and magnitude of the upshift vary between experiments. However, compatible solutes have a significant influence, a role that the MOPS could assume in our medium (27). The initial response of cell elongation or volume growth after osmotic upshifts has been reported to occur within a few minutes (31–33), similar to what we observed in microfluidics for cell length. The time course of the crowding transition of 1 to 2 h is in the range of biopolymer synthesis (and changing the proteome) and proceeds throughout the cell division stage. Therefore, even though cell elongation resumes rapidly after shock, a new proteome needs to be synthesized to arrive at a new crowding homeostasis over longer periods. The change in crowding may be assisted by the biosynthesis of, e.g., trehalose, which has been reported to accumulate to maximum values in up to an hour in E. coli cells grown in medium without compatible solutes (34). Although the DNA content can increase under hyperosmotic stress (35), especially under higher osmotic shocks than we use, we do not consider the DNA to be a classical crowder due to its large size and immobility. However, DNA might have an indirect effect by reducing the total available volume for other crowders or act through confinement mechanisms. Together, the kinetics of cell length and crowding suggest that the crowding changes are initially governed by cell volume, after which the cytoplasm arrives at a new state through biopolymer synthesis.

Although we consider an increase in the ribosome fraction of the total biopolymer content to be a likely source of the decrease in effective macromolecular crowding, other phenomena could contribute. For example, we show here that energy dissipation decreases the effective excluded volume as well, which is an effect that occurs within 2 min, which is too fast for major changes in the proteome or/and ribosome content. Here, we achieve perhaps a state where the cytoplasm is more gel-like or colloidal glassy and thus leaves more uncrowded spaces for the probes to occupy (36). Moreover, even if the sensor and the cytoplasm were homogeneously mixed, crowder self-associations would decrease the excluded volume effect of the crowders (11). Such a state could be enhanced by the absence of ATP that potentially acts as a hydrotope and solubilizes the proteome (29). Hence, different biochemical states of the cytoplasm could yield the same effective excluded volume.

Cells adapt to external stress to maintain cell growth. We mapped the changes in macromolecular crowding during adaptation to an osmotic upshift, a condition previously shown to alter the biochemical organization of the cell. We show that the cells indeed arrive at a new state where the effect of the excluded volume is decreased, which may be caused by alteration in the particle size distribution in the cytoplasm or change in biochemical organization (Fig. 4). This would provide a mechanism to adopt higher-biopolymer-volume fractions while maintaining an effective crowding homeostasis with excluded volume effects tuned by the particle size and/or mobility.

## MATERIALS AND METHODS

### Cell growth and confocal imaging.

Cell preparation, growth, and imaging were performed as described previously (19, 22). Briefly, the pRSET A vector containing the synthetic gene encoding either crGE, crE6G2, or crG18 was transformed into E. coli BL21(DE3). The cells were incubated at 30°C, with shaking at 200 rpm, in 10 ml MOPS medium (pH 7.2) with 20 mM glucose and grown overnight. The next day, the cells were diluted into 50 ml of fresh medium to an OD_600_ of 0.05. When the OD_600_ reached 0.1 to 0.2, the cells were imaged. Subsequently, the concentration of NaCl was raised to 300 mM by the addition of prewarmed 3 M NaCl in MOPS medium, and the cells were imaged to follow their recovery. The OD_600_ was monitored over time, and the cell culture was diluted with prewarmed medium (MOPS medium plus 300 mM NaCl) to reduce the OD_600_ every time from 0.3 to 0.1. For imaging, a 0.5-ml sample of cells expressing one of the probes was combined with 0.5 ml of cells that contained monovalent streptavidin in pRSET A (blank). A parallel culture was maintained under the same conditions to provide the blank cells. The combined cells were 10× concentrated by centrifugation and resuspended. Subsequently, 10 μl of the cells was transferred to a glass slide modified with (3-aminopropyl)triethoxysilane and imaged on a laser scanning microscope (LSM; Zeiss 710). The probes were excited using a 405-mm LED, and the emission was split into 450- to 505-nm (mCerulean3) and 505- to 797-nm (FRET) channels. The fluorescence intensities for each cell were determined in ImageJ, and background originating from the blank cells was subtracted.

### Imaging in a microfluidics chamber.

The microfluidic chamber (CellASIC Onix microfluidic plates) was prewarmed overnight at 30°C on the laser scanning confocal microscope. The E. coli BL21(DE3) cells with desired crowding probe and the control strain containing monovalent streptavidin were grown overnight to an OD_600_ of 0.1 to 0.3, which is still in the exponential-growth phase, and then diluted to an OD_600_ of 0.01 in MOPS-glucose minimal medium and subsequently loaded in the microfluidic chamber. After loading, the cells were incubated with 0.1× MOPS-glucose medium (MOPS-glucose medium diluted 10× with 0.16 M NaCl) at 30°C for 2 h. After 2 h, the medium in the microfluidics was replaced by 0.1× MOPS-glucose medium that contained an additional 0.3 M NaCl on top of the 0.16 M. Alternatively, we used 600 mM and 1 M sorbitol instead of the 0.3 M NaCl for osmotic upshift in the microfluidic chamber. The images were collected and analyzed as described above.

### Cell volume determination.

The volume of the cytoplasm was determined by photoactivated localization microscopy (PALM). The gene encoding LacY was fused to YPet which can switch “on” or “off” during imaging in PALM (37). The gene encoding LacY-YPet was cloned into the pACYC vector and transformed into E. coli BL21(DE3). The cells (inoculated from a single colony) were grown at 30°C with shaking at 200 rpm in 10 ml MOPS medium with 20 mM glucose overnight. The next day, at an OD_600_ of 0.2, the cells were induced with 0.1% l-rhamnose. One hour after induction, the cells were imaged by PALM before and after the addition of 300 mM NaCl.

Coverslips were cleaned with 5 M KOH in a sonication bath for 30 min and washed with demineralized water and acetone (Aldrich). Next, the coverslips were plasma-cleaned for 10 min and subsequently coated with 2% (vol/vol) (3-aminopropyl)trioxysilane (Aldrich) in acetone for 30 min. The coverslips were washed with demineralized water and left to dry.

For PALM, a home-built inverted microscope based on an Olympus iX81 microscope with a high-numerical-aperture (NA) objective (×100, NA = 1.49, oil immersion, UApo) was used. Solid-state lasers were from Coherent (Santa Clara, CA) at 514 nm (Sapphire 514, 100 mW). Imaging was performed in semi-total internal reflection fluorescence (semi-TIRF) mode with the angle of light exiting the objective adjusted to create a light sheet restricted to the bottom few micrometers of the specimen. The fluorescence was recorded using an electron-multiplying charge-coupled-device (EM-CCD) camera from Hamamatsu, Japan, model C9100-13. For data acquisition and analysis, LacY-YPet was continuously illuminated at 517 nm, and 3,000 frames were recorded with 30 ms for each frame. The data were analyzed with a home-written ImageJ script, in which the reconstructed images of each fluorescent molecule are represented as a single spot at its determined coordinates, with a brightness that corresponds to the localization accuracy (37).

### Preparation of cell lysate.

The E. coli BL21(DE3) cells were incubated in 10 ml MOPS with 20 mM glucose at 30°C and shaking at 200 rpm overnight and then diluted to 1 liter of fresh medium to an OD_600_ of 0.02. When the OD_600_ reached 0.2, half of the culture was lysed immediately, while the other half was lysed after incubation for 5 h with 300 mM NaCl. To lyse the cells, the cultures were harvested by centrifugation (3,000 × *g*, 30 min). The pellet was resuspended in 10 mM sodium-inorganic phosphate (NaPi) with 100 mM NaCl (pH 7.4), containing Mini cOmplete, EDTA-free proteinase inhibitor. Cells were lysed by sonication for 2 min, with alternating 5 s of sonication and 5 s of cooling, and then centrifuged (20,000 × *g*, 10 min). The supernatant was immediately used for the fluorescence measurements.

### Fluorometry.

Fluorescence emission spectra were measured with a Fluorolog-3 spectrofluorometer (Jobin Yvon). A 1.0-ml solution (10 mM NaPi, 100 mM NaCl, 2 mg/ml bovine serum albumin [BSA] [pH 7.4]) was added to a quartz cuvette, and its fluorescence emission spectrum was recorded after excitation at 420 nm (spectrum A). Subsequently, purified sensor was added and mixed by pipette, and the fluorescence was recorded (spectrum B). The desired amount of small molecule or cell lysate was added and mixed by pipette, and the fluorescence was recorded again (spectrum C). The background spectrum A, prior to addition of the probe, was subtracted from spectrum B or C.

### OD_600_ measurements during FCCP treatment.

A 96-well plate (Greiner) was filled with four replicates of each of the different culture or condition combinations (200 μl/well), with the remaining wells filled with either filter-sterilized MOPS-glucose minimal medium or Milli-Q (MQ) water. The plate was covered with gas-permeable film and mounted on a plate reader (BioTek PowerWave 340). While shaking the plate at 30°C, the absorbance at 600 nm was read every minute for 3.5 h using the Gen 5 software. After the plate reader measurement, the OD_600_ values were referenced to the average of the absorbance values for MQ water for each specific time point.

## Supplementary Material

Supplemental file 1
