# Supplementary material for: Decreased Effective Macromolecular Crowding in Escherichia coli Adapted to Hyperosmotic Stress
Source: J Bacteriol. 2019 Apr 24;201(10):e00708-18. doi: 10.1128/JB.00708-18 (PMC6482933; doi:10.1128/JB.00708-18)
Supplement: Supplemental file 1 [file JB.00708-18-s0001.pdf]

Supplemental Material

Decreased Effective Macromolecular Crowding in *Escherichia coli* Adapted to Hyperosmotic Stress

Boqun Liu,[a,b] Zarief Hasrat,[a] Bert Poolman,[a,c]# Arnold J. Boersma[a,d]#

[a] Department of Biochemistry, Groningen Biomolecular Sciences and Biotechnology Institute, University of Groningen, Groningen, The Netherlands

[b] Jilin Provincial Key Laboratory of Nutrition and Functional Food, Jilin University, Changchun, People's Republic of China

[c] Zernike Institute for Advanced Materials, University of Groningen, Groningen, The Netherlands

[d] DWI-Leibniz Institute for Interactive Materials, Aachen, Germany

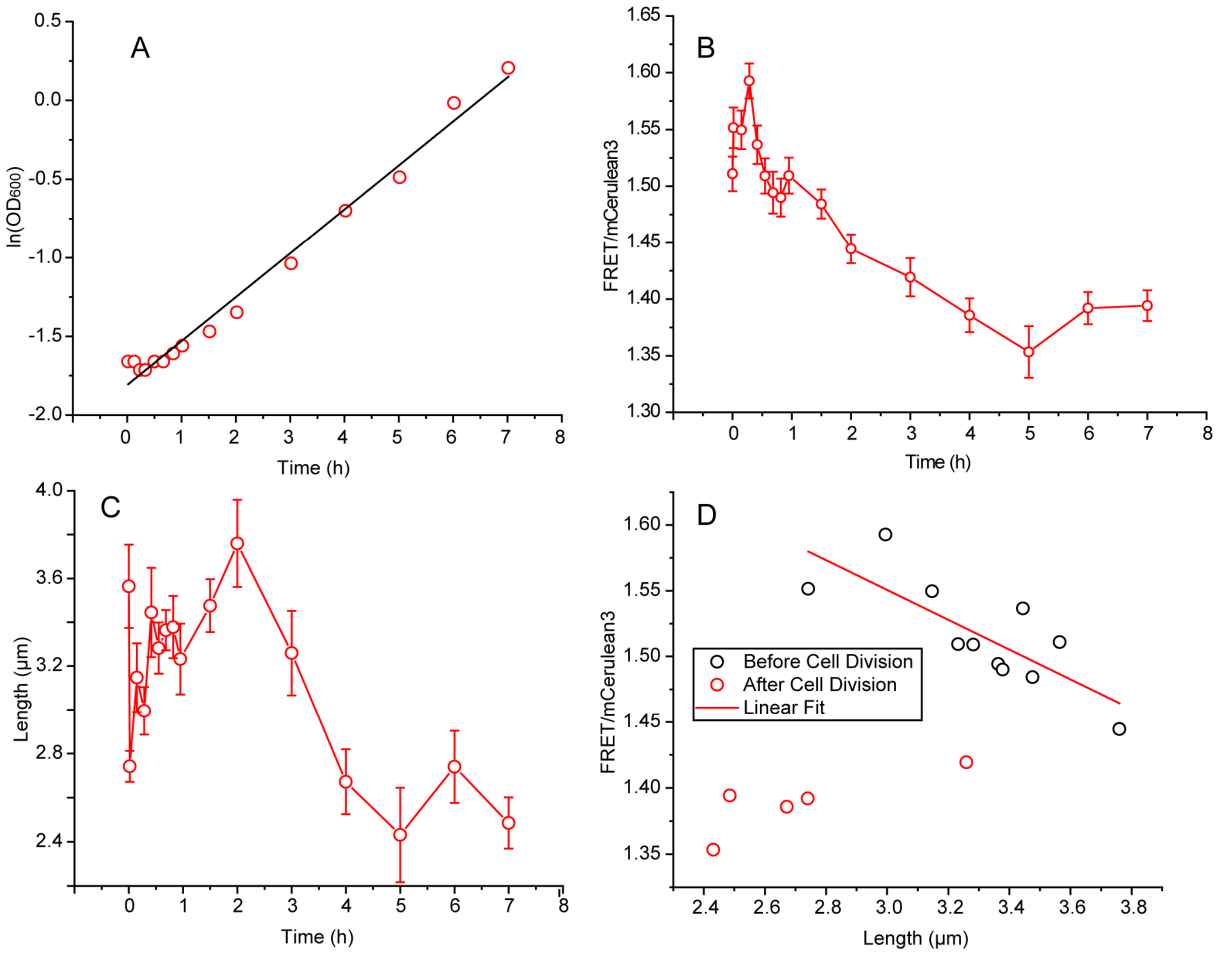

Figure S1. Response to 300 mM NaCl of *E. coli* BL21(DE3) equipped with the crG18 probe in pRSET A, as in Figure 1. **A**. The *ln*OD_{600} decreases after the addition of 300 mM NaCl to MOPS-glucose medium and subsequently increases linearly over time, passing the pre-upshift OD_{600} after ~1h. The OD_{600} is corrected for continuous dilution of the culture to maintain the OD_{600} between 0.1 and 0.3. The data fits a linear with R^2=0.99, indicating a stable growth rate. Technical error is 0.01. A single biological replicate is displayed. **B**. The FRET/mCerulean3 ratio of the crG18 sensor present in the *E. coli* cells as measured by confocal fluorescence microscopy. The ratios immediately increase upon osmotic upshift and decrease after one hour to levels lower than prior to the osmotic upshift. All data is for at least 60 *E. coli* cells and error bars reflect standard error. **C**. Osmotic upshift results in a decrease in median cell length as measured by fluorescence microscopy (same cells as in panel B), which is followed by an increase in length of the synchronized cells until division starts, resulting in smaller cells compared to pre-upshift conditions. All data is for at least 60 cells and error bars reflect standard error. **D**. Data from panel B and C combined showing the relation (linear approximation: R^2=0.62) between the FRET/mCerulean3 ratio and the median cell length (black circles), which holds until the cells divide. After that, the FRET/mCerulean3 remains low (red circles).

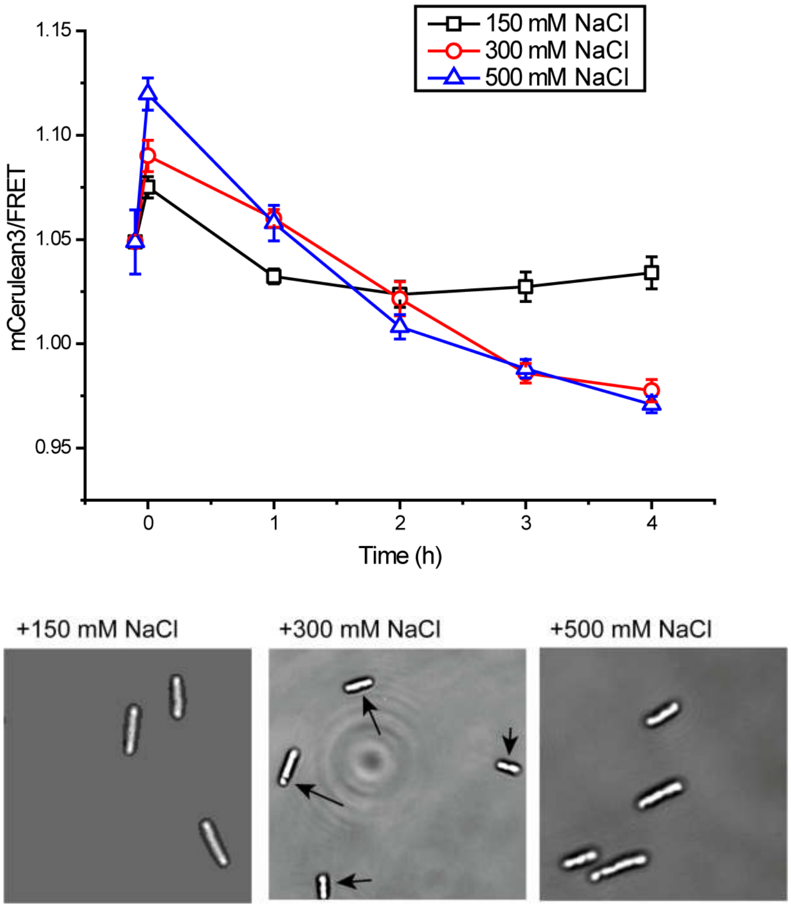

Figure S2: Adaptation of *E. coli* BL21(DE3) to osmotic stress as probed by the crGE crowding sensor in pRSET A. Cells were incubated in MOPS-glucose at 30 °C in a batch culture. At t = 0, the cells were treated with different concentrations of NaCl. Errors bars show the standard error over ~100 cells. Bottom panels are brightfield images of the cells immediately after the corresponding upshift. Arrows point to invaginations that are occasionally found with osmotic upshifts of 300 and always with 500 mM NaCl.

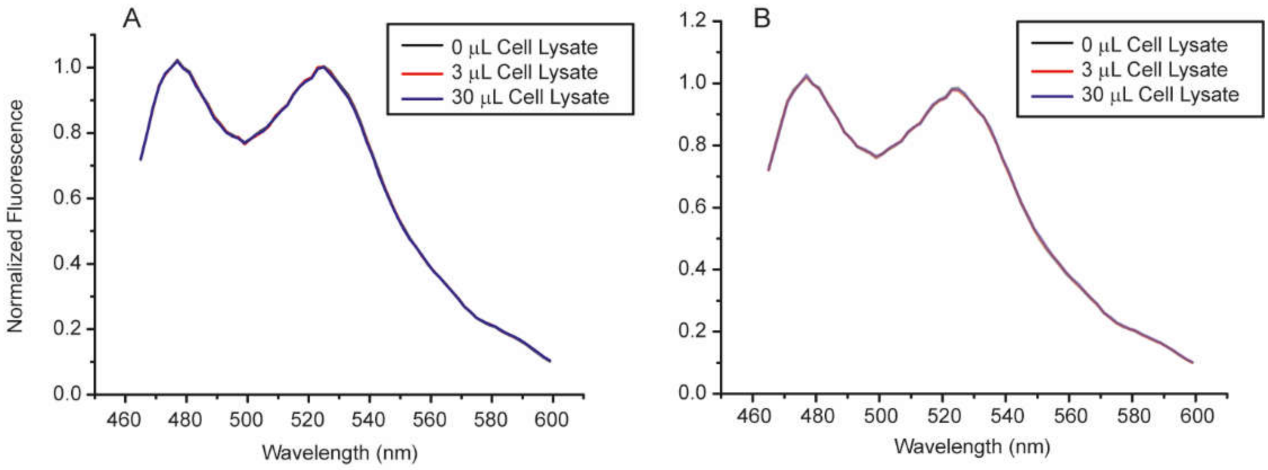

Figure S3: Fluorescence spectra of crG18 in the absence and presence of lysate. The crG18 was dissolved in 10 mM sodium phosphate (NaPi), 100 mM NaCl, pH 7.4 and titrated with different volumes of concentrated cell lysate (20 mg of total protein/ml). A: titration with cell lysate from cells that were harvested before osmotic upshift. B: titration with cell lysate from cells that were harvested 5 h after an osmotic upshift with 300 mM NaCl.

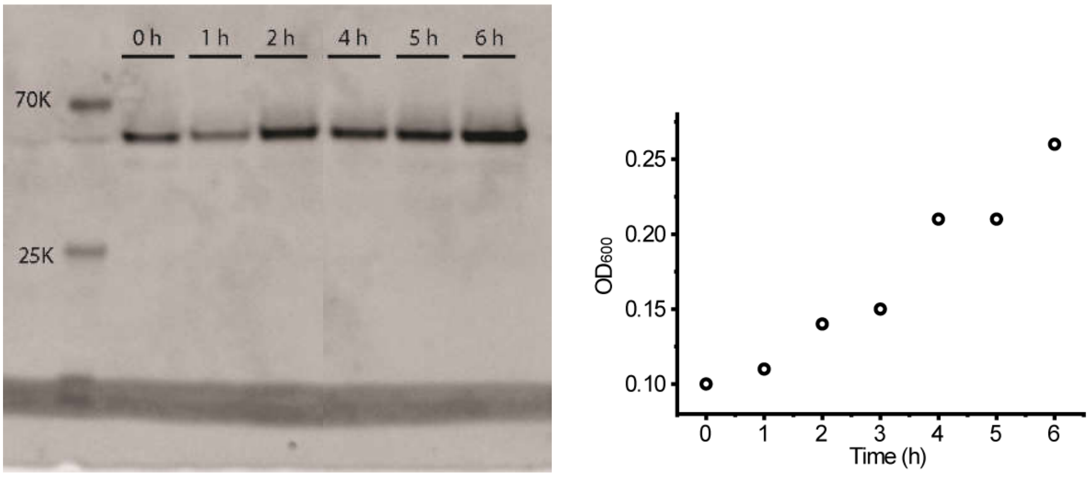

Figure S4. In-gel fluorescence of crGE isolated from *E. coli* BL21(DE3) at various stages of growth and adaptation to osmotic stress. The cells expressing crGE were incubated in MOPS-glucose medium at 30 °C and shaking at 200 rpm. The cells were subjected to a 500 mM NaCl osmotic upshift. The cells were lysed with Bugbuster 10X (Novagen®) and loaded onto a 12 % SDS-PAGE gel. The 3h time point was removed due to incomplete cell lysis. The increasing intensities are caused by the increasing OD_{600} displayed in the right panel. The amount of loaded protein was not corrected for the OD_{600}.

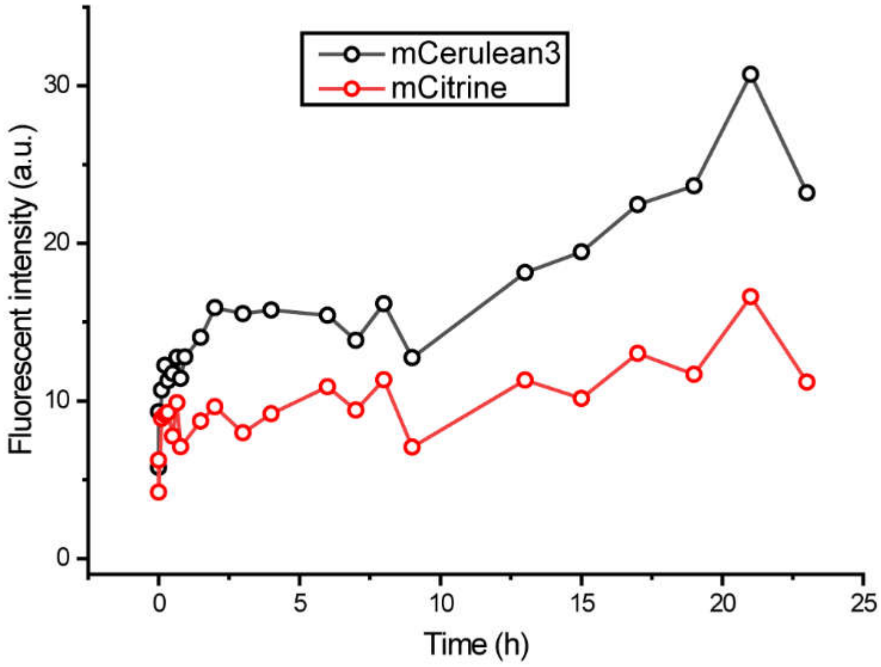

Figure S5: Fluorescence of mCitrine and mCeruline3 present in crGE during adaptation of *E. coli* BL21(DE3) to a 300 mM NaCl upshift (at t=0) obtained by fluorescence confocal microscopy; the cells were grown in MOPS-glucose medium. The data correspond to the experiment shown in Figure 1. Black circles are the median intensity of mCerulean3 in crGE, obtained by LED excitation at 405 nm and emission at 450 – 505 nm, while red circles are the median intensity of mCitrine, obtained by laser excitation at 488 nm and emission at 505 – 798 nm. Data is a single biological replicate from at least ~60 cells per data point and is based on the cells shown in Figure 1.

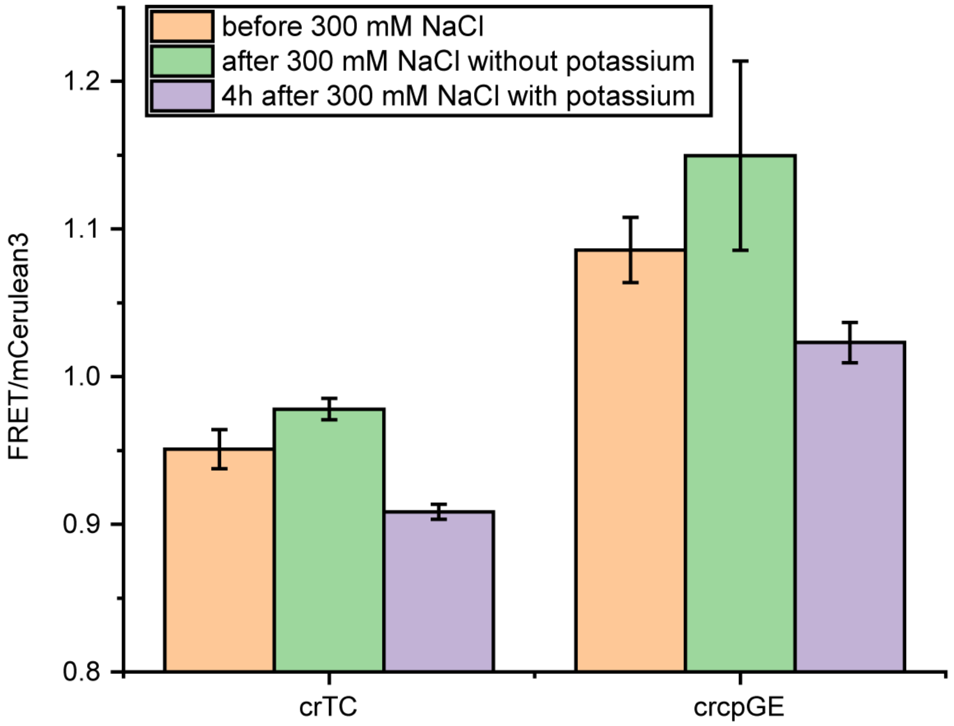

Figure S6: Comparison of different FRET-based crowding probes, in the pRSET A plasmid present in BL21(DE3). The crTC probe contains mTurquoise2 and mCitrine, while the crcpGE contains mCerulean3 and cpmVenus. The data recorded in MOPS-glucose medium (brown bar), MOPS medium with 300 mM NaCl but without potassium and glucose to prevent recovery (green bar), and incubated for 4h in MOPS-glucose with 300 mM NaCl (purple bar). Data is the average and standard deviation over three independent experiments.

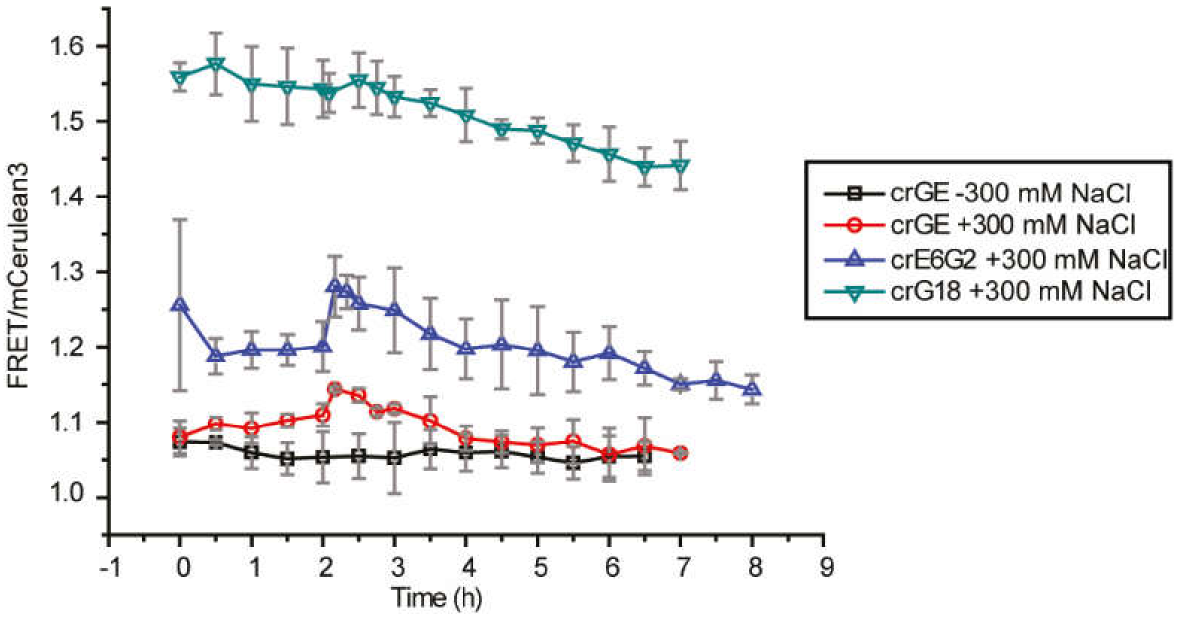

Figure S7: Response of different sensors expressed in *E. coli* BL21(DE3) and analyzed in the microfluidic chamber. Cells were grown in the chamber in 0.1× MOPS-glucose plus 0.16 M NaCl, which has an osmolality similar to MOPS-glucose. At 2 h, the same medium supplemented with 300 mM NaCl was flowed in. The black squares show the data of cells that were not subjected to an osmotic upshift. Error bars show the standard deviation from three independent biological replicates, each based on ~100 cells.

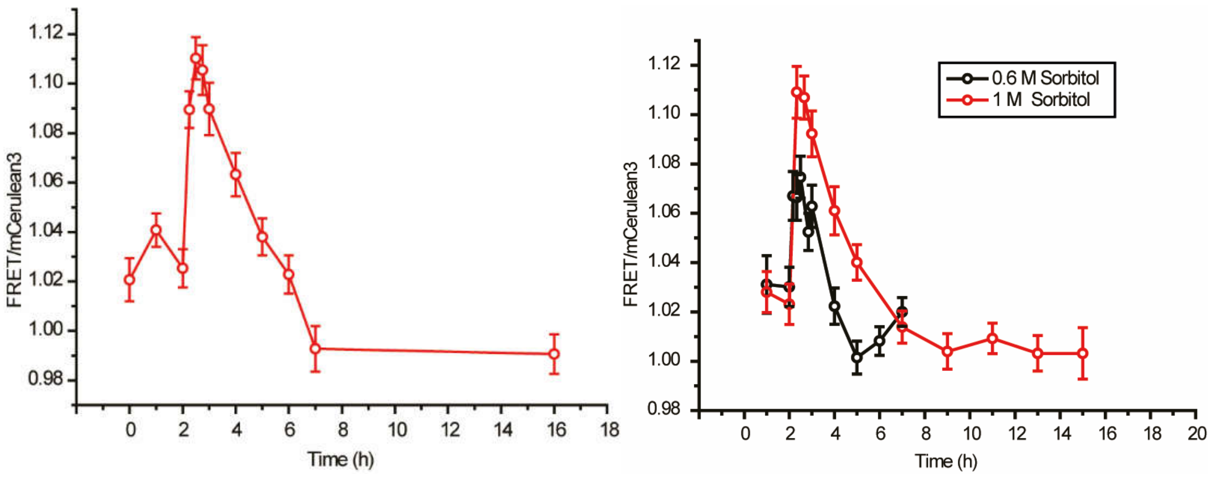

Figure S8: Crowding changes in *E. coli* BL21(DE3) cells upon osmotic upshift (500 mM NaCl or 0.6-1M sorbitol) and analyzed in the microfluidics devices. Top panel: Cells expressing crGE were loaded into a microfluidic chamber at t = 0 and incubated at 30 °C in 0.1x MOPS-glucose medium with 160 mM NaCl. At t = 2h, 0.1x MOPS-glucose with 500 mM NaCl was flowed in. Bottom panel: Cells expressing crGE sensors were treated the same as A. At = 2h, 0.1x MOPS-glucose medium with the desired concentration sorbitol were flowed in. Error bars are standard error over ~100 cells.

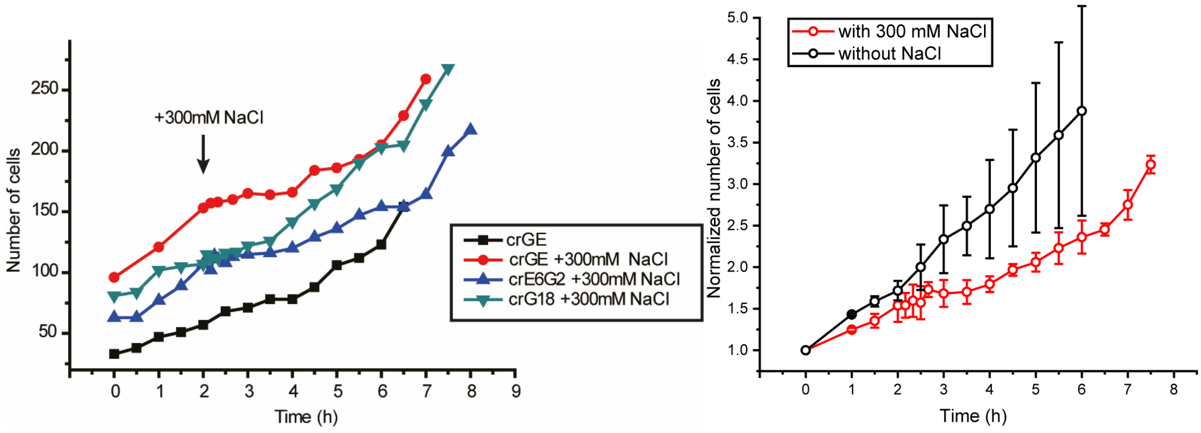

Figure S9. Left panel: Increase in absolute number of cells in microfluidics. Right panel: Normalized number of cells. Error bars are standard deviation over three biological replicates. Image size was 531×106 μm. Same conditions as Fig S7 apply.
